# Polymer Composite Sandwich Panels Composed of Hemp and Plastic Skins and Composite Wood, Recycled Plastic, and Styrofoam Cores

**DOI:** 10.3390/polym17101359

**Published:** 2025-05-15

**Authors:** Ashiqul Islam, Wahid Ferdous, Paulomi (Polly) Burey, Kamrun Nahar, Libo Yan, Allan Manalo

**Affiliations:** 1 School of Engineering, Centre for Future Materials (CFM), University of Southern Queensland, Toowoomba, QLD 4350, Australia; ashiqul.islam@unisq.edu.au (A.I.); allan.manalo@unisq.edu.au (A.M.); 2 School of Agriculture and Environmental Science, Centre for Future Materials (CFM), University of Southern Queensland, Toowoomba, QLD 4350, Australia; polly.burey@unisq.edu.au (P.B.); kamrun.nahar@unisq.edu.au (K.N.); 3 Department of Organic and Wood-Based Construction Materials, Technische Universität Braunschweig, Hopfengarten 20, 38102 Braunschweig, Germany; l.yan@tu-braunschweig.de

**Keywords:** sandwich panels, hemp skins, PET skins, composite wood, waste plastic, styrofoam cores

## Abstract

This paper presents an experimental investigation of six different types of composite sandwich panels manufactured from waste-based materials, which are comprised of two different types of skins (made from hemp and recycled PET (Polyethylene terephthalate) fabrics with bio-epoxy resin) and three different cores (composite wood, recycled plastic, and styrofoam) materials. The skins of these sandwich panels were investigated under five different environmental conditions (normal air, water, hygrothermal, saline solution, and 80 °C elevated temperature) over seven months to evaluate their durability performance. In addition, the tensile and dynamic mechanical properties of those sandwich panels were studied. The bending behavior of cores and sandwich panels was also investigated and compared. The results indicated that elevated temperatures are 30% more detrimental to fiber composite laminates than normal water. Composite laminates made of hemp are more sensitive to environmental conditions than composite laminates made of recycled PET. A higher-density core makes panels more rigid and less susceptible to indentation failure. The flexible plastic cores are found to be up to 25% more effective at increasing the strength of sandwich panels than brittle wood cores.

## 1. Introduction

The lightweight, strong, and stiff nature of composite sandwich panels has drawn a great deal of attention in aerospace, automotive, and construction applications where being lightweight, high performance, and resource efficient are crucial [1,2,3]. The sandwich panels consist of two skins separated by a thick core, offering a versatile solution. By strategically combining materials, these panels enhance structural strength and stiffness while addressing essential factors such as energy efficiency and cost-effectiveness.

There are various types of commercially available sandwich panels, and each panel is designed for specific applications. A common type of skin is a fiber-reinforced polymer (FRP), such as carbon fiber-reinforced polymer (CFRP), glass fiber-reinforced polymer (GFRP), or aramid fiber-reinforced polymer (AFRP) [4,5]. These materials provide high strength-to-weight ratios and excellent fatigue resistance. A metal skin, particularly aluminum or stainless steel, can also withstand harsh environments due to its durability [6]. In certain applications, thermoplastic skins like polypropylene and polyethylene are versatile and cost-effective [7]. Materials for the cores are equally diverse, with lightweight foam cores such as polyurethane, polystyrene, and polyvinyl chloride (PVC) providing good thermal insulation [8,9,10]. Aluminum, Nomex (aramid), and paper honeycomb cores offer exceptional strength and rigidity while remaining lightweight [11,12]. Cores made of wood, especially balsa wood, are valued for their stiffness and low density [13,14]. Furthermore, corrugated cores, made of plastic or metal, provide structural support and are frequently used in the construction and packaging industries [15,16]. In addition, in relation to the geometry and the orientation of the corrugated core, the Kirigami corrugated cores have good impact resistance, 3D-printed bi-directional corrugated cores are better than conventional cores, trapezoidal corrugated cores are good shock absorbers, axial/circular corrugated cores have good impact resistance characteristics, and trapezoidal, rectangular, and triangular cores have a higher energy absorption ability [16]. Through the combination of these skins and cores, sandwich panels can be customized to meet the performance, weight, durability, and cost requirements of various industries. While these materials provide good structural performance, their environmental sustainability in terms of carbon emissions remains an issue.

In recent years, there have been several attempts to develop low-emission sandwich panels. Researchers [17] investigated the bio-based sandwich beams made from paper honeycomb core filled with foam and flax fiber-reinforced composite skin with different orientations. This type of sandwich panel meets some structural requirements, but these panels are not available on the market yet. Another researcher [18] studied balsa wood core and cellulose fabric bio-based polypropylene composite skins; however, their bio-based PP contained only around 30% bio-based content. Moreover, another experimental study was carried out with the corrugated cardboard core sandwich beams with bio-based flax fiber composite skins having different orientations of core and skins for large-scale building applications [19]. The mechanical properties of sandwich panels manufactured from recyclates, flax fiber, and bio-based epoxy for semi-structural applications were studied [20]. Another researcher [21] studied aluminum sheet skins, a gapping bamboo ring core, and castor oil biobased adhesive, and the outcome showed that the 40% increase in the internal void by bamboo ring cores reduced the maximum load and flexural strength by 63% and 59%, respectively, and the team has suggested, for future research, small gaps of bamboo rings and large dimensions, which will ensure the bending conditions. In addition, some other researchers tested Tetra Pak waste as a core and aluminum face sheets as skins [22]. Moreover, the waste tire rubbers with polyester resin and four layers of jute fabric laminate as skins were used to manufacture the sandwich panels [23]. In the case of bio-resin use in sandwich panels, the bio-polyurethane (bio-PU) adhesive made from the castor oil plant was used in sandwich panels, the core was waste rubber with Portland cement, and the skins were aluminum faces [24]. Overall, the above researchers attempted to produce low-emission panels; they can further be improved by incorporating 100% natural bio-based and waste-based materials.

Recently, several initiatives, including a net-zero emission plan and circular economy, have been taken by world leaders to minimize global carbon emissions. Australian legislation aims to achieve net-zero emissions by 2050, where by 2030, 43% of emissions will be reduced to below 2005 levels [25]. Another proposed roadmap of the Australian government shows that 80% of all types of waste materials will be recovered by 2030 [26]. Also, circular economy initiatives are crucial to achieving net-zero emissions, so construction materials should be included in these initiatives [27]. Therefore, construction materials should be manufactured using green alternatives and waste-based materials in order to meet all of the objectives.

In this study, three different waste-based core materials were combined with natural and recycled plastic fabric skins, along with bio-based resins. This approach aimed to meet both performance and environmental requirements, including low-carbon emissions and adequate mechanical strength. The carbon emissions of the materials were studied to assess the carbon footprint of the proposed material. The tensile and dynamic mechanical properties of skins and bending properties of cores and sandwich panels were evaluated in order to gain a better understanding of their behavior. The outcome of this study will contribute to the development of sustainable sandwich panels today and in the future.

## 2. Materials and Methods

### 2.1. Materials

#### 2.1.1. Bio-Epoxy Resin

In this study, a 100% bio-based epoxy resin was utilized to manufacture the laminates and binders of sandwich panels. Bio-epoxy resin is a non-toxic, recyclable resin created from a by-product of biodiesel [28]. The pH of the bio-resin was 6–8, and the thermal decomposition temperature was 180 °C. This bio-epoxy can be used for structural adhesives. Bio-epoxy resin consists of two components, part A and part B, with densities of 1.24 kg/L and 0.93 kg/L, respectively, as well as a mixing ratio of 75% to 25% (by weight). The viscosity of part A resin is 750 cP (centipoise), the viscosity of part B resin is 15 cP at 25 °C, and the mixed viscosity is 150 cP at 25 °C.

#### 2.1.2. Fabric

The two types of skin materials used were hemp fabric and PET (Polyethylene terephthalate) fabric. A hemp fabric is a fabric made from natural hemp fibers grown in Australia, which is primarily used in composite reinforcement applications. The hemp fabric had a simple woven pattern and was uniform in its strength. The density of lightweight, strong, and 100% biodegradable hemp fabric is about 135 g/m^2^ [29]. Similarly, PET fabric made from recycled PET polyester has a woven pattern and is uniform in strength, with a density of about 105 g/m^2^ [29].

#### 2.1.3. Waste-Based Core Materials

The three types of selected core materials were composite wood, recycled plastic panel, and recycled styrofoam, which were manufactured by COEN, Replas products, and polystyrene products [30,31,32] in Brisbane, Australia. The composite wood core material was made up of 60% recycled wood flour, 30% recycled HDPE plastic, and 10% binding agent. The composite wood was engineered for use in structural applications such as decking, cladding, and fencing [33]. The recycled plastic cores were made from waste plastics and primarily used for decking bollards and fencing. Recycled plastic products have numerous benefits, including being environmentally friendly, low maintenance, termite resistant, and long lasting [32]. The recycled styrofoam cores were made from waste styrofoam, are eco-friendly, and can be customized in density and size [31]. The densities of composite wood, recycled plastic, and recycled styrofoam were approximately 1300 kg/m^3^, 900 kg/m^3^, and 15 kg/m^3^, respectively. The waste-based core materials were selected to reduce the carbon emissions associated with the sandwich panels, thereby aligning with the objectives of the experiment.

### 2.2. Methods

#### 2.2.1. Manufacturing of Composite Materials

##### Manufacturing of Skins

**Figure 1:**
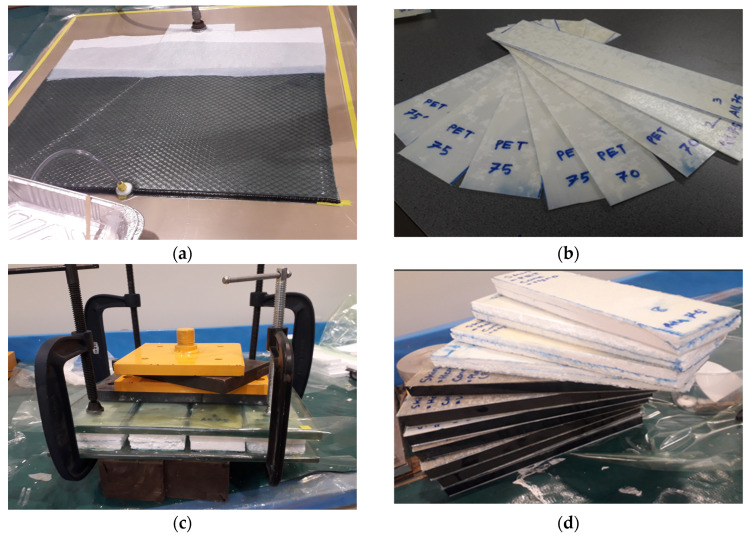
Composite laminate preparation: (**a**) vacuum bagging process; (**b**) typical PET skin laminates; (**c**) manufacturing of sandwich panels; (**d**) typical sandwich panels.

The vacuum bagging process was used to manufacture the skins of sandwich panels. The vacuum bagging process has the capability to produce superior performance and high-quality composite materials. Five layers of fabric with 0° orientation were placed in a vacuum bag to achieve the desired skin thickness, as shown in Figure 1a,b. The resin was then infused into the vacuum bag under a pressure of approximately 14.7 psi, converting the soft fabric layers into high-quality laminates [34]. Laminates are cured in an oven at 100 °C for one day. In general, hemp and PET (Polyethylene terephthalate) composite laminates are approximately 900 mm long and 400 mm wide, with a 2 mm thickness and a 35% fiber and 65% bio-epoxy resin content (after trial mixing process). The full laminates were cut to the desired sizes using a water jet cutter. Skins were exposed to normal air and four extreme environmental conditions, including water, hygrothermal (60 °C temperature and 98% humidity), 10% concentrated saline water, and 80 °C elevated temperature. In this research, only the skins were chosen for environmental testing, as the outer surface of sandwich panels is typically the one that faces severe environmental impact. A period of seven months was considered for weathering sandwich skins.

##### Manufacturing of Sandwich Panels

Two skins including hemp and PET (Polyethylene terephthalate) were cut to specific dimensions along with three core types, including composite wood (350 mm long, 75 mm wide, 25 mm thick), recycled plastic panel (350 mm long, 70 mm wide, 25 mm thick), and recycled styrofoam (350 mm long, 75 mm wide, 25 mm thick), using similar dimensions to the relevant core sandwich panel. Bio-epoxy resin was used to adhere to the desired size cores and skins. The resin was first injected onto the rough skin surface, and film applicators were used to ensure even coverage; then, it was placed onto the bottom glass, and the core was then gently placed on the skin. Secondly, the rough side of the other skin was chosen for spreading resin and placed on the core. Finally, the top glass was set, and clamps and weights were placed in a systematic manner. As the base and top plates, two 25 mm thick glass plates were used, and four clamps and a 20 kg steel weight were used to apply uniform and adequate pressure to the paneling system so that consistent bonding could be ensured across the skin and core interface. The panels were subjected to pressure for approximately one day. A total of six different types of panels were prepared.

#### 2.2.2. Testing of Skins

##### Tensile Test of Skins

The ASTM D3039 testing standard was followed to test 10 different types of tensile samples (2 skins and 5 environments) [35]. Five samples of each type of composite skin were tested. The samples were cut into a dog-bone shape to ensure the failure at the mid-span. Over the course of seven months, the skins were exposed to five different environmental conditions (normal air, water, hygrothermal, 10% concentrated saline water, and elevated temperatures to simulate a normal environment, rainwater, a warm and humid environment, a coastal area or marine environment, and a high heat environment, from the sun, respectively). For each type of laminate, five replicate samples were tested. The tensile test result of the skin was obtained using the MTS 10 kN capacity testing machine (manufactured in Tokyo, Japan). All samples were tested at a speed of 2 mm/min. The appropriate data collection process was implemented to collect the required information.

##### Dynamic Mechanical Analysis (DMA) of Skins

The glass transition temperature of the composite laminate was measured with NETZCH 242 E Artemis (produced in Yatala, Australia) according to ASTM D4065 [36]. The DMA test of skins was conducted to know the failure temperature of the polymer composite. Eight different types of samples were prepared from hemp and PET (Polyethylene terephthalate) laminates (i.e., two skins with four environments). There were two replicate samples of each type in four different environmental conditions (i.e., normal air, water, hygrothermal, and 10% concentrated saline water). The dimensions of the samples were 45 mm long by 10 mm wide by 2 mm thick, cut by water jet. The laminates were placed in a configuration and loaded at a frequency ranging from room temperature to 150 °C with a ramp rate of 5 °C/min. Storage modulus (E′), loss modulus, and tan delta data were recorded and plotted against temperature.

#### 2.2.3. Bending Test of Cores

The three different core samples, each with three replications, were tested in accordance with ISO 14125 [37]. Three-point bending tests were conducted to determine the core’s bending capacity. The testing was conducted on an MTS 100 kN testing machine (manufactured in Tokyo, Japan) with a 2 mm/min test speed to test the core sample with a span length of 300 mm.

#### 2.2.4. Bending Test of Sandwich Panels

Six types of sandwich panels were manufactured (i.e., two skins and three cores) with three replicate samples of each type. The dimensions were approximately 350 mm long, 75 mm wide for wood and styrofoam cores and 70 mm for plastic cores, and 29 mm thick. Tests were conducted on all sandwich panels under three-point bending on an MTS testing machine with a capacity of 100 kN, a support span of 300 mm, and a test speed of 2 mm/min. The strain gauges were installed at the bottom of the mid-span in order to measure the maximum bending strain of the panels.

## 3. Results and Observation

### 3.1. Failure Mode

#### 3.1.1. Failure Mode of Skins

**Figure 2:**
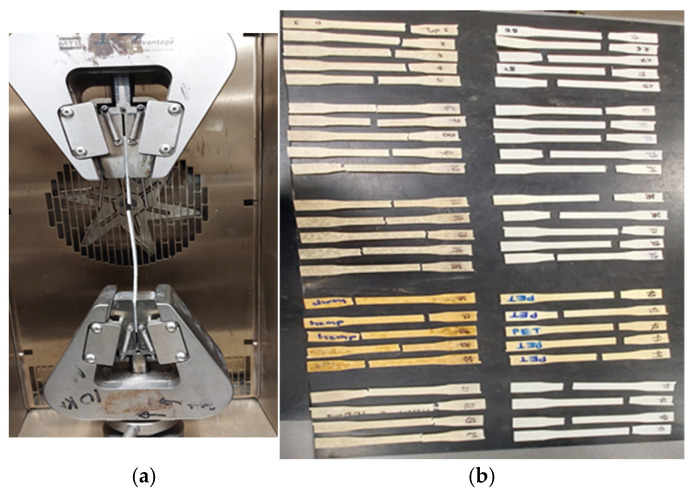
Tensile sample failure patterns of composite skin. (**a**) Tensile test setup. (**b**) All skin samples tested after 7 months (hemp skin, left side; PET skin, right side, with five different environments, i.e., normal air, water, hygrothermal, saline solution, and elevated temperature from top to bottom sequence).

Under uniaxial loading, the tensile test revealed the failure mechanisms for the sample composite skins. An example of a typical skin failure following tensile testing on five-layered composite skins can be seen in Figure 2. The failure of the specimens was observed in the tensile span zone, demonstrating the specimens’ pure tensile capacity. The tab location did not appear to be damaged.

#### 3.1.2. Failure Modes of Cores

**Figure 3:**
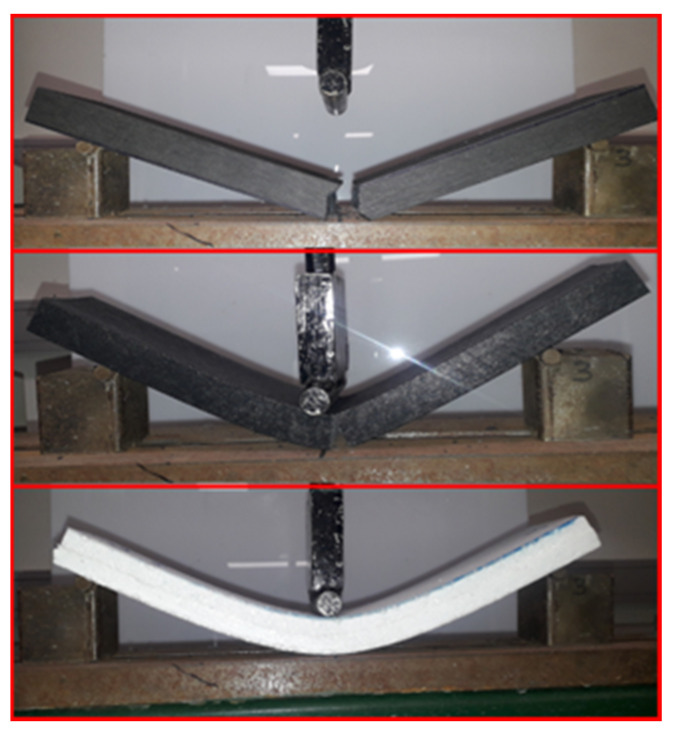
Failure pattern of cores (wood, plastic, and styrofoam, respectively).

Different failure modes were observed as a result of the use of different cores, as depicted in Figure 3. An abrupt failure of the wood core samples occurred at the mid-span, representing the brittle nature of the failure. The plastic core deflected much higher than the wood core before failure occurred at mid-span. This is due to the composition of the material, which is a combination of different plastics, resulting in shorter polymer chains, thereby causing greater deformation under stress. The styrofoam cores were able to deflect rapidly at low loads as a result of their easily compressible cellular structure and relatively low density.

#### 3.1.3. Failure Modes of Sandwich Panels

**Figure 4:**
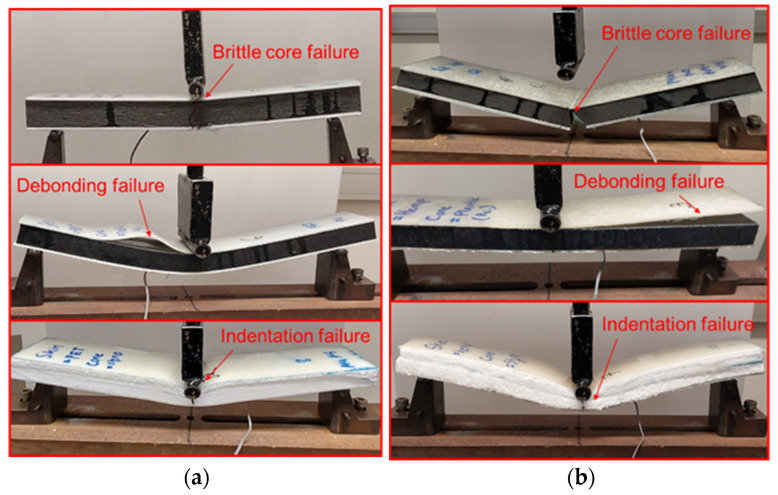
Failure pattern of sandwich panels: (**a**) PET skin sandwich panels. PET skin with wood composite core, PET skin with plastic core, and PET skin with styrofoam core sandwich panel (top to bottom, respectively). (**b**) Hemp skin sandwich panels. Hemp skin with wood composite core, hemp skin with plastic composite core, and hemp skin with styrofoam core sandwich panel (top to bottom, respectively).

Different failure modes were observed among sandwich panels based on the variations in cores and skins (Figure 4). The brittle composite wood core panel failed at mid-span, which caused the bottom skins to fail prematurely. Therefore, it is essential to design the skin properly in order to be able to effectively utilize its strength for brittle cores. Sandwich panels with a plastic core failed due to debonding between the skins and the core. Due to the high deflection of the plastic core, there is a high horizontal shear stress between the skin and the core during bending, causing the skin to debond from the core. This indicates that, in the case of flexible cores, it is essential to take into account the bond strength between the skin and the core. Sandwich panels with a styrofoam core failed due to indentation, due to the variations in load-resisting capacity between the compressible cellular structure of the core and the skin. Therefore, lightweight styrofoam cores may not be appropriate for sandwich panels with high-stress concentrations.

### 3.2. Tensile Stress–Strain Behavior of Skins

**Figure 5:**
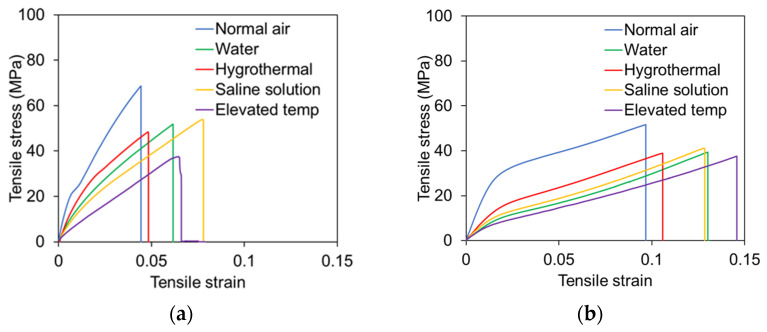
Variation in tensile stress–strain relationship of skin of sandwich panels in different environments. (**a**) Hemp skin; (**b**) PET skin.

All tensile samples of skins were loaded uniaxially until failure. Figure 5a,b show that the tensile strength decreased under different environmental conditions. The average ultimate tensile strength of hemp skins was 60 MPa in normal air, 52 MPa in water, 48 MPa in hygrothermal conditions, 48 MPa in saline solution, and 35 MPa in 80 °C elevated temperature, as illustrated in Figure 5. The stress–strain curve of hemp skins was linear, and sudden failure occurred as shown in Figure 5a. For the PET skin, the average ultimate tensile strengths for normal air, water, hygrothermal conditions, saline solution, and elevated temperature were 50 MPa, 40 MPa, 40 MPa, 45 MPa, and 39 MPa, respectively. According to Figure 5b, the tensile stress–strain plot showed linear behavior until a certain stress and strain level and then showed non-linear behavior with gradually increasing stress and strain values, resulting in sudden failure.

### 3.3. Dynamic Mechanical Analysis (DMA) of Skins

**Figure 6:**
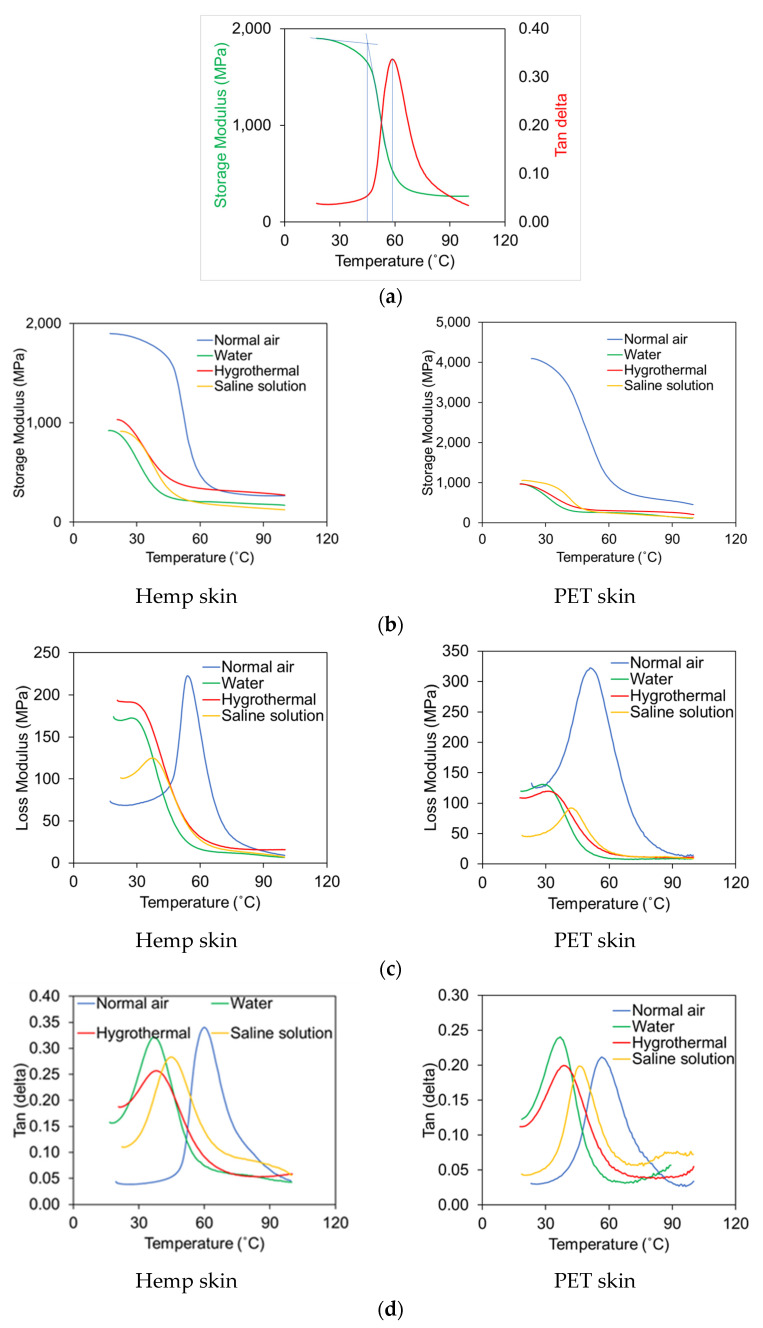
Variation in dynamic mechanical properties with temperature. (**a**) Typical DMA plot (ASTM D7028); (**b**) storage modulus vs. temperature (hemp left side and PET right side); (**c**) loss modulus vs. temperature; (**d**) tan delta vs. temperature.

The graph in Figure 6 illustrates a typical DMA graph, with each sample displaying three glass transition temperatures that can be estimated from the tan delta, storage modulus, and loss modulus. There is a clear difference in the glass transition temperature (Tg) between the tan delta, storage modulus, and loss modulus curves. Determining the glass transition temperature of the storage modulus involves drawing two tangents. The first tangent starts from the beginning point of the glass transition curve, while the next one begins between the inflection point and the approximate mid-point of the drop of the storage modulus curve. The tan delta Tg value was measured from the peak of the curve, where the value was slightly higher than the Tg of the storage modulus. However, various studies consider different Tg values. For instance, the ASTM D7028 [38] test standard refers to both values of Tg, while other researchers [39,40,41] suggest evaluating the Tg from the peak of the tan delta curve. Conversely, the ASTM D4065 [36] standard suggests obtaining the Tg from the peak of the loss modulus curve.

The Tg of hemp skin for storage modulus and loss modulus ranged from 45 °C to 55 °C in normal air. When hemp skin was placed in various environments, the Tg values for the storage modulus and loss modulus ranged from 25 °C to 40 °C. The Tg value for hemp skin in normal air was found to be 60 °C in the case of tan delta and ranged from 35 °C to 45 °C under varying environmental conditions. The Tg values of PET skin ranged from 38 °C to 50 °C for both the storage modulus and loss modulus cases, and in various environmental types, the Tg values ranged from 25 °C to 45 °C. The tan delta curve of PET skins provided a Tg value of 57 °C, and in varied environmental conditions, the Tg value varied from 35 °C to 45 °C. Table 1 provides a summary of the skin results.

### 3.4. Bending Properties of Core Materials

**Figure 7:**
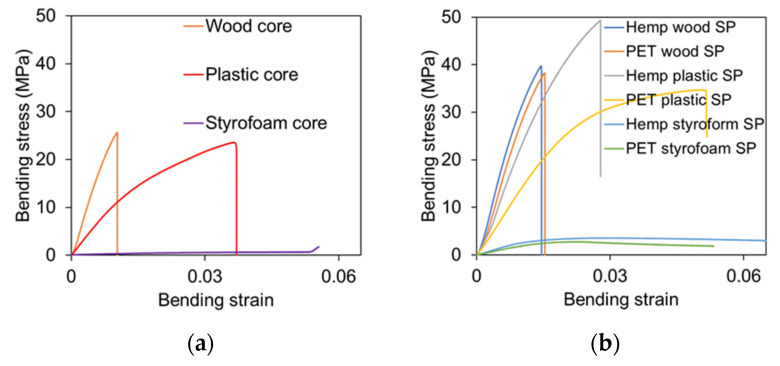
Bending behavior of cores and sandwich panels. (**a**) Flexural stress–strain relationship of cores; (**b**) flexural stress–strain relationship of sandwich panels (SP: sandwich panels).

The stress–strain behavior of the sandwich core materials is presented in Figure 7a. The wood core, being brittle, exhibited a linear response, while the plastic cores showed slightly non-linear behavior, and the soft, lightweight styrofoam displayed a linear yet highly flexible response. As a result of this study, the average bending stress of the wood core, the highly flexible plastic core, and the soft and lightweight styrofoam core were found to be about 28 MPa, 28 MPa, and 1 MPa, respectively. The density of materials appears to play a significant role in determining their bending strength. The structure of wood composites and plastics is usually more compact and has stronger intermolecular forces, resulting in a higher degree of bending resistance compared with styrofoam, which is less dense and has weaker intermolecular forces.

### 3.5. Bending Properties of Sandwich Panels

The stress–strain behavior of the sandwich panels is shown in Figure 7b. The bending stress–strain curves for the sandwich panels were plotted using the wood, plastic, and styrofoam cores with specific additions of hemp and PET skins. The maximum bending stress of wood core hemp skin sandwich panels, plastic core hemp skin sandwich panels, and styrofoam core hemp skin sandwich panels were 39 MPa at strain 0.014, 49 MPa at strain 0.027, and 3 MPa at strain 0.025, respectively. On the other hand, the bending stress of wood core PET skin sandwich panel, plastic core PET skin sandwich panel, and styrofoam core PET skin sandwich panels were 38 MPa at strain 0.015, 36 MPa at strain 0.053, and 3 MPa at strain 0.025, respectively. A full summary of the average bending results of cores and sandwich panels is shown in Table 2.

## 4. Discussion

### 4.1. Effect of Environmental Conditions

**Figure 8:**
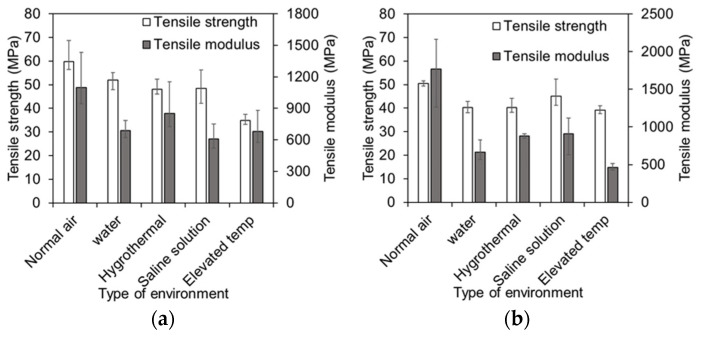
Effect of environmental conditions on skin material. (**a**) Hemp skins; (**b**) PET skins.

The skins were tested for tensile strength and glass transition temperature after conditioning for seven months in five different environments. In normal air, hemp skin samples have a tensile strength of 60 MPa, which decreased by 13%, 20%, 20%, and 42% when exposed to normal water, hygrothermal, saline water, and elevated temperatures, respectively. Moreover, the tensile modulus of hemp skin in normal air is 1099 MPa, whereas the tensile modulus of hemp skin samples under different environmental conditions decreased, such as 37% in water, 22% in hygrothermal conditions (60 °C temperature and 98% humidity), 44% in a saline solution environment, and 38% in elevated temperature conditions (Figure 8). The glass transition temperature (Tg) of hemp skin is higher in normal air than in the samples under different types of environmental conditions, as shown by the storage modulus, loss modulus, and ten delta plots. The Tg of tan delta of hemp skin samples in normal air is 60 °C, whereas the Tg value under different environmental conditions is decreased, such as 42% in water samples, 37% in hygrothermal samples, and 25% in saline solution samples, as shown in Table 1.

Tensile properties and glass transition temperature analysis reveal that the skins can be affected by weather conditions. Water absorption typically reduces fiber and matrix strength, which leads to the degradation of the mechanical and thermodynamic properties of laminates. A hygrothermal environment was created in which the skins were placed in a chamber with a temperature of 60 °C and a relative humidity of 98%. Before characterizing the environmental impact of the specimens, the specimens were visually inspected. When the resin is exposed to hygrothermal conditions, it swells, softens, or undergoes hydrolytic degradation, weakening its bond with the fibers. As a result, the stiffness, strength, and dimensional stability can be reduced. Moisture absorption increases at elevated temperatures, which speeds up degradation. Moisture can also cause plasticization of the resin, resulting in a lower glass transition temperature (Tg), which causes the resin to soften sooner. High moisture can also weaken fibers themselves and lead to debonding of fibers and matrixes. This combination of elevated temperature and moisture significantly reduces the durability and mechanical performance of skin materials (i.e., 22% of the tensile strength and 37% of the glass transition temperature were reduced for hemp skin). When exposed to saline environments (10% salinity), the polymer matrix can swell, plasticize, and degrade. Moisture absorption weakens the interfacial bonding between fibers and the matrix, reducing tensile strength, stiffness, and structural integrity. Moreover, prolonged exposure to saline conditions can accelerate chemical degradation processes, such as hydrolysis, further compromising the composite’s durability. Tensile tests of skin were conducted at an elevated temperature of 80 °C. When the resin is heated, it becomes softer, and degradation mechanisms become more active. Moreover, the tensile strength of hemp skin is reduced by 42% at elevated temperatures because the Tg of the tan delta is 60 °C, which is lower than the 80 °C elevated temperature.

### 4.2. Effect of Skin Materials

The hemp and PET (Polyethylene terephthalate) skins were utilized in the study, and the behavior of these skins was revealed by conducting the tensile tests and dynamic mechanical analysis. Overall, the tensile strengths of natural fabric hemp skins under different environments are affected more than the PET skins. In normal air, hemp skin has a tensile strength of 50 MPa and PET skin has a tensile strength of 60 MPa, while their tensile moduli are 1.1 GPa and 1.77 GPa, respectively. Despite this, both hemp and PET skins exhibit similar glass transition temperatures (Tg), as shown in Table 1. The performance of natural laminate hemp skin compared to PET skin in sandwich panels can be affected by several factors. Biodegradable hemp skins possess a lower mechanical performance than synthetic PET skins. The Tg of PET is determined by the mobility of the polyester backbone, whereas hemp fibers have a Tg determined by the relaxation of the amorphous regions within the cellulose matrix. Aside from this, recycling PET fibers may contain structural changes as a result of processing and degradation, which lead to chain scission and cross-linking, leading to a Tg similar to that of natural hemp laminates.

### 4.3. Effect of Core Materials

**Figure 9:**
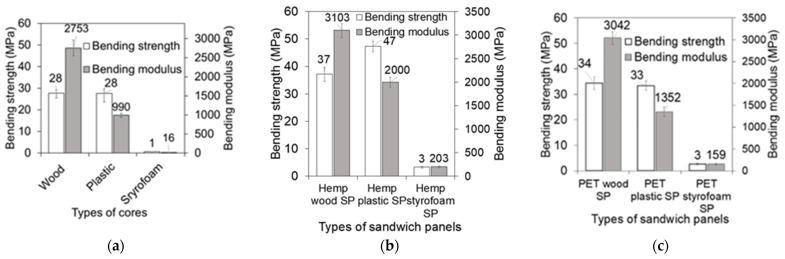
Bending test results of sandwich panels. (**a**) Cores only; (**b**) hemp skin sandwich panels; (**c**) PET skin sandwich panels.

The core materials have a significant impact on the behavior of the sandwich panel, as results shown in Figure 9. After manufacturing the sandwich panels, the bending strength of wood cores with hemp and PET skins rose by 32% and 21%, respectively. Similarly, the bending strength of composite plastic cores with hemp and PET skins increased by 68% and 18%, respectively. In terms of styrofoam cores, the bending strength improved by 200% for both hemp and PET skins. The hemp skin–composite wood core sandwich panels failed with less bending strain compared to the hemp skin–plastic core sandwich panels due to the stiff nature of the wood core. The hemp skin–wood core sandwich panels bending strength and strain are 18% and 40% less, respectively, than the hemp skin–plastic core sandwich panels, as shown in Figure 7. The reason for this is that the failure occurred in the core for a skin–wood core sandwich panel, whereas the plastic core was able to withstand significant deformation before failing.

The density of the cores plays an important role in determining how they bend. There is a general observation that panels with a high core density are generally stiffer. Wood composites, plastic, and styrofoam cores have densities of 1300 kg/m^3^, 900 kg/m^3^, and 15 kg/m^3^, respectively. Increasing the density of the core improves the material’s ability to transfer shear forces between the face sheets, thereby reducing core deflection. When a low-density styrofoam core is used in a design, greater shear deformation occurs, adversely affecting the face sheet’s ability to distribute the load over the core. Due to excessive compression at the loading point, the core’s yield strength is exceeded, which leads to the indentation failure of the sandwich panels made from styrofoam. The use of recycled styrofoam core sandwich panels can meet a variety of structural requirements, regardless of these challenges, especially in non-load-bearing or low-impact environments. Due to the lightweight insulation and buoyancy provided by the styrofoam core, these panels are ideal for applications that require low weight and high thermal efficiency.

## 5. Theoretical Modeling for the Prediction of Failure Loads

An analysis of the theoretical modeling for the prediction of failure loads was conducted in order to estimate the maximum load that the sandwich panel could withstand before failure. The load capacity of the sandwich beams was determined based on experimentally observed failure modes. These are core bending failures for composite wood core panels, skin debonding failures for recycled plastic core panels, and indentation failures for styrofoam core panels.

### 5.1. Brittle Wood Core Panel Failure Load

**Figure 10:**
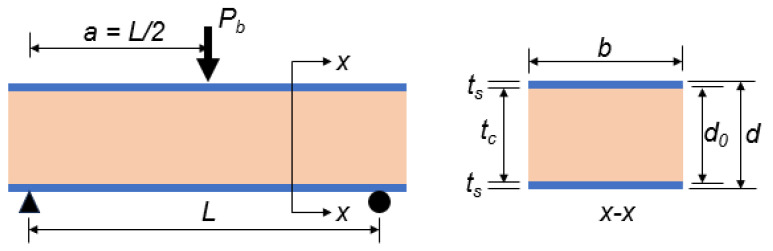
Loading arrangement and sectional dimensions.

It is anticipated that the sandwich panels will fail during bending when the core’s bending stress ($\sigma_{c}$) approaches its maximum value. In order to determine the ultimate failure load ($P_{b}$) resulting from the bending failure of a flatwise oriented sandwich panel (Figure 10), Equation (1) should be used.

(1) $$P_{b}=\frac{4\left(EI\right)\sigma_{c}}{adE_{s}}$$

Equation (2) can be used to calculate the theoretical bending stiffness (EI) in a flatwise orientation.

(2) $$EI=\frac{bt_{c}^{3}}{12}E_{c}+\frac{bt_{s}}{2}\left(\frac{t_{s}^{2}}{3}+d_{0}^{2}\right)E_{s}$$

where $E_{s}$ and $E_{c}$ are the skin and core modulus, respectively. The contribution of the core to bending stiffness can sometimes be ignored in order to make the theoretical estimation more conservative.

### 5.2. Flexible Plastic Core Panel Failure Load

In flatwise orientation, the sandwich panels are expected to fail in the skin due to the high deflection of the core. The ultimate failure load resulting from bending loads ($P_{b}$) of flatwise sandwich panels can be determined using Equation (3).

(3) $$P_{b}=\frac{4\left(EI\right)\sigma_{s}}{adE_{s}}$$

where $\sigma_{s}$ is the strength of the skins.

### 5.3. Soft and Lightweight Styrofoam Core Panel Failure Load

It is expected that soft and lightweight styrofoam core sandwich panels will fail in indentation when oriented flatwise. The ultimate failure load of flatwise sandwich panels resulting from bending loads ($P_{b}$) can be determined by Equation (4).

(4) $$P_{b}=\frac{4}{3}kbt_{s}\sqrt{\sigma_{c}\sigma_{s}}$$

In this equation, k represents the support coefficient. In many cases, the load-carrying capacity can be reduced by as much as 60% when switching from an elastic foundation to simply supported conditions due to the loss of continuous support. Therefore, the value of k is 1 if the beam is supported on an elastic foundation, while it is taken to be 1/3 if it is simply supported [42].

### 5.4. Comparison Between Experimental and Theoretical Failure Load

The load–displacement relationship is used to evaluate the bending stress and bending modulus experimentally, as shown in Table 1 and Table 2. The experimental failure stresses are determined from the laboratory data. Theoretically, the bending failure load of sandwich panels in flatwise orientation can be estimated using Equations (1)–(4).

**Figure 11:**
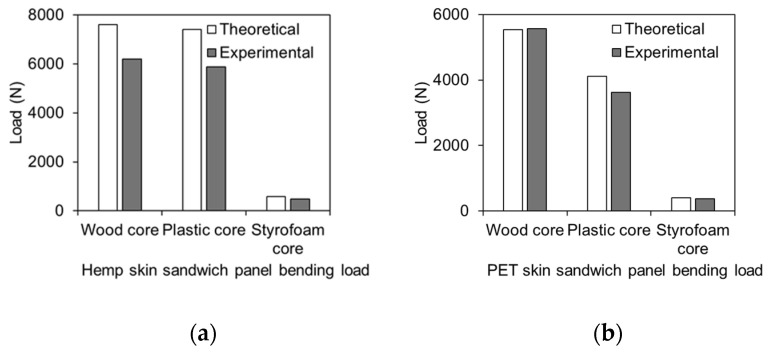
Comparison between experimental and theoretical failure loads of the sandwich panels. (**a**) Hemp skin panels failure load; (**b**) PET skin panels failure load.

Figure 11 illustrates a comparison between the experimental and theoretical failure loads. The theoretical estimation of failure load is slightly higher than the corresponding experimental loads. This is perhaps due to the consideration of core stiffness in Equation (2), where the bending loads of the beam are usually carried by skins. The results indicate that the analytical equations are capable of reasonably estimating the actual failure load of the sandwich panels.

## 6. Conclusions

This study investigates the mechanical and durability properties of two types of skins (hemp and recycled PET) and three different types of waste-based cores (wood composites, recycled plastics, and styrofoam), incorporating six types of sandwich panels. The tensile behavior and glass transition temperatures of the skins are studied under normal air, water, hygrothermal, saline solution, and elevated temperature conditions. The bending behavior of the cores and sandwich panels is investigated, and the capacity of the panels is predicted using theoretical modeling. Based on the results, the following conclusions are drawn:

- Temperature is found to be more detrimental to fiber composite laminates than other environmental conditions (water, hygrothermal, saline solution, and normal air). The reason for this is that elevated temperatures soften the polymers of the skins, which results in a faster loss of mechanical properties than other environments.
- Hemp skins are more sensitive to different environmental conditions than recycled PET skins. While hemp skins lost up to 40% of their tensile strength, PET skins lost around 20% due to aggressive environments. However, both hemp and recycled PET skins drop their glass transition temperatures quite similarly, by 35%. Hemp skins degrade faster than recycled PET skins due to their higher water absorption, as hemp is a natural fiber.
- The stiffness of the core plays an important role in the bending behavior of sandwich panels. A stiffer core improves the material’s ability to transfer shear forces between the face sheets, thereby reducing core deflection. Higher core stiffness makes panels less likely to fail from indentation.
- The type of core has a significant impact on the theoretical prediction of the failure load of sandwich panels. The strength of the core dominates the load capacity in brittle core sandwich panels, while the strength of the skin dominates the load capacity in flexible core sandwich panels. In low-stiffness cores, the load capacity is dependent upon the resistance to core indentation.

This study aims to develop sustainable sandwich panels created from natural and landfill waste materials in order to reduce landfill waste, achieve net-zero emissions goals, and create a circular economy. Based on the preliminary results of the investigation, it appears that the goal may be achievable. A dynamic impact, fatigue, large-scale structural behavior, and free vibration study, including a simulation of panels under different environments, will further confirm the viability of this technology.

## Figures and Tables

**Table 1:** Summary of skin results.

Environmental Condition	Tensile Strength (MPa)		Tensile Modulus (MPa)		Tg from Tan Delta (°C)	
	Hemp	PET	Hemp	PET	Hemp	PET
Normal Air	60	50	1099	1772	60	57
Water	52	40	690	662	35	35
Hygrothermal	48	40	852	880	38	38
Saline solution	48	45	611	909	45	45
Elevated temp.	35	39	681	462	-	-

**Table 2:** Summary of bending strength and bending modulus of cores and sandwich panels.

Core Types	Core Average Results		Sandwich Panel Average Results			
			Hemp Skin		PET Skin	
	Strength (MPa)	Modulus (MPa)	Strength (MPa)	Modulus (MPa)	Strength (MPa)	Modulus (MPa)
Wood	28	2753	37	3103	34	3042
Plastic	28	990	47	2000	33	1352
Styrofoam	1	16	3	203	3	159

## Data Availability

The original contributions presented in this study are included in the article. Further inquiries can be directed to the corresponding author.
